# Study of Child Labor Among School Children in Urban and Rural Areas of Pondicherry

**DOI:** 10.4103/0970-0218.40881

**Published:** 2008-04

**Authors:** K Devi, Gautam Roy

**Affiliations:** Department of Preventive and Social Medicine, Jawaharlal Institute of Postgraduate Medical Education and Research (JIPMER), Puducherry, India

## Introduction

Child labor is broadly defined as any form of economic activity for at least 1 hour per week and/or domestic chores for at least 7 hours per week and/or school labor for at least 5 hours per week.(1)

According to estimates, in developing countries alone there are 250 million children in the age group of 5-17 years who are toiling in economic activity - i.e., one out of every six children in the world today. In absolute terms, it is Asia (excluding Japan) that has the most child workers (approximately 61% of the world's total).(2) A study done in Pondicherry determined that 15% of children in the urban school in Pondicherry were engaged in some form of economic work.(3) The policy appears to have little impact on the situation, as poverty is deep rooted and compels children to work.(4) Hence the complex issue of child labor and its ramification is worth investigating. It was strongly felt that children who work and attend school could have some disadvantage compared to school children who are not engaged in work. It was therefore decided to carry out the present study on working children who attend school, as it was felt that they may have special problems of having to cope with the burden of studies and work.

## Objectives

1) To determine the prevalence of child labor among school children in the rural and urban areas of Pondicherry; and 2) To study the factors related to child labor - like the reasons for working, problems faced by the child, workplace conditions, etc.

## Materials and Methods

The study was carried out in the schools situated in the service areas of Jawaharlal Institute Rural Health Center (JIRHC) and Jawaharlal Institute Urban Health Center (JIUHC). The JIRHC and JIUHC are the rural and urban field practice areas of Jawaharlal Institute Postgraduate Medical Education and Research Center (JIPMER), Pondicherry. It was decided to conduct the study among students in classes VI to X.

For the purpose of the study, child labor was defined as any kind of work done by a school-going child for remuneration in cash or kind. For calculating the sample size, the average prevalence of school-going child labor was taken as 35% (50% in rural areas and 15% in urban areas). Using the formula 4PQ/L(2) the required sample size was estimated to be 743, rounded off to 750.

## Procedure

The principals of the schools selected from the service area were contacted, and the purpose of the study was explained to them in detail. Permission was then obtained from the Director of Education, Pondicherry, to conduct the study in selected schools of the rural and urban areas of Pondicherry.

The questionnaire and the interview schedule were first tested among 10 students of another school, not in the service area. After making a few modifications based on the responses obtained, the questionnaire was finalized. To attain the required sample size, all the students enrolled in classes VI to X of the two schools in the JIUHC service area were included. In the schools of the JIRHC service area, lots were used to decide which classes were to be included. There were 759 eligible students in the classes of the selected schools, and only 720 students (414 urban and 306 rural) could be contacted. The children who were working were further interviewed using a pre-tested interview schedule. Interview was conducted for the working children alone in their respective houses with the help of the identification data collected in the questionnaire.

Chi-square test and t-test were used to find out the association between the attributes. Logistic regression analysis was done to find the adjusted odds ratio for the selected risk factors using SPSS software (SPSS version - 13).

## Results

The overall prevalence of child labor in the study was 32.5%. The number of students who worked in the rural and urban area was 131 (42.8%) and 103 (24.9%) respectively.

Irrespective of the area, educational level of the mother, crowding in the family, families being in debt, presence of a handicapped or alcoholic member in the family, gender and religion were significantly associated with the working child [Table 1].

**Table 1:** Logistic regression model of risk factors for having to go to work

Variable	B	SE	Wald	df	Sig	Exp (B) (95% CI)
Boys vs girls	1.317	0.194	45.999	1	0.000	3.73 (2.55-5.46)
Not living with family vs living with family	−0.292	0.394	0.548	1	0.459	7.47 (0.35-1.62)
Hindu vs others	0.792	0.319	6.168	1	0.013	2.21 (1.18-4.12)
No formal education of father vs some formal education	−0.167	0.233	0.515	1	0.473	0.85 (0.54-1.34)
No formal education of mother vs some formal education	0.545	0.194	7.889	1	0.005	1.73 (1.18-2.52)
Handicapped member in family vs no handicapped member	1.123	0.568	3.904	1	0.048	3.07 (1.01-9.36)
Alcoholic member in family vs no alcoholic member	0.727	0.190	14.594	1	0.000	2.07 (1.43-3.01)
Debt in family vs no debt in family	1.022	0.207	24.383	1	0.000	2.78 (1.85-4.17)
Overcrowding vs no overcrowding	0.525	0.236	4.964	1	0.026	1.69 (1.07-2.69)
Socio-economic Class V vs other classes	0.652	0.338	3.729	1	0.053	1.92 (0.99-3.72)
Constant	−8.696	1.719	25.577	1	0.000	0.000

Z = (−8.696) + (1.317) gender + (0.792) religion + (−0.292) living with family + (−0.167) father's education + (0.545) mother's education + (1.123) handicapped member + (0.727) alcoholic member + (1.022) debt + (0.525) overcrowding + (0.652) lower socioeconomic class

Ninety percent of the children in the rural area and 80.8% in the urban area said low income was the main reason for them to go to work. Overall, 78.6% visited a health facility like a health center or hospital in the past 1 year for any health complaints. About 75.9% of the rural working children reported that their employer scolded them at the workplace. The proportion of working children who were scolded by their employer at the workplace in urban area was 87.2%. In the rural area, 65.1% of the working children were beaten or scolded by their employer for working slowly. Similarly in the urban area, 62.8% of the working children were beaten or scolded for slow work.

## Discussion

The study revealed that 32.5% of children went to work. In the rural area, the proportion of students who worked was 42.8%; in the urban area, the corresponding proportion was 24.8%. From a community-based study conducted among school children in Nigeria, Bolanle M Fetuga *et al.* found that 64.5% of the school-going children were engaged in work.(5) Nitin *et al.* in a cross-sectional study found that prevalence of child labor among the slum children in Nagpur was 21.3%.(6) A community-based cross-sectional study among school children in Pondicherry found the prevalence of child labor to be 15% among school children.(3) The differences in the prevalence may reflect the differences in methodology and mode of data collection and the lack of standard definition of child labor. In the informal sector of the economy, the magnitude of working children is virtually unknown because many of the establishments are not registered with the proper government regulatory agencies.

In the present study, it was observed that more children from families from the lower socioeconomic stratum (i.e., Class V of the Modified Kuppuswamy score) went to work. Nitin *et al.* in their study in Nagpur found that a lower socioeconomic status of the family was significantly associated with child laborers.(6) The present study showed that irrespective of whether the children came from families in poverty or otherwise, in the rural areas the children went to work. This may also be because the quantification of the income of the parents in the rural area is difficult; and therefore it appears that regardless of the total household income, the child has to engage in some work. The present study revealed that in both the rural and urban areas, working children spent less time studying as compared to their nonworking counterparts. Nivethida *et al.* in their study from the same urban area found that half of the children felt that their work affected their studies.

Logistic regression analysis showed that children coming from families in debt had 2.78 times the risk of having to go to work compared to those from debt-free families. Presence of a handicapped member or an alcoholic in the family put the child at 3.07 and 2.07 times the risk respectively of having to go to work compared to there being no such member in the family. Children who came from overcrowded families had a higher risk of having to go to work. Children of mothers who had no formal school education had 1.73 times the risk of being sent to work compared to those of mothers who had formal school education. In the rural and urban areas where the study was conducted, the living conditions were more or less the same, and hence there was not much difference in the risks associated with the working status of a child.
